# Complete Genome Sequence of *Bacillus thuringiensis* Serovar Israelensis Strain HD-789

**DOI:** 10.1128/genomeA.01023-13

**Published:** 2013-12-05

**Authors:** Norman A. Doggett, Chris J. Stubben, Olga Chertkov, David C. Bruce, J. Chris Detter, Shannon L. Johnson, Cliff S. Han

**Affiliations:** Biosecurity and Public Health Group, Bioscience Division, Los Alamos National Laboratory, Los Alamos, New Mexico, USAa; Bioenergy and Biome Sciences Group, Bioscience Division, Los Alamos National Laboratory, Los Alamos, New Mexico, USAb

## Abstract

*Bacillus thuringiensis* is an important microbial insecticide for controlling agricultural pests. We report the finished genome sequence of *Bacillus thuringiensis* serovar israelensis strain HD-789, which contains genes encoding 7 parasporal crystals consisting of Cry4Aa3, Cry4Ba5 (2 genes), Cry10Aa3, Cry11Aa3, Cry60Ba3, and Cry60Aa3, plus 3 Cyt toxin genes and 1 hemagglutinin gene.

## GENOME ANNOUNCEMENT

*Bacillus thuringiensis* strain HD-789 was obtained from the USDA Agricultural Research Service (Peoria, IL), and the genome sequence was generated using a combination of Illumina (1) and 454 (2) technologies. The whole-genome sequence was obtained from three Illumina GAii shotgun libraries which generated 49,827,921 reads totaling 5,331.6 Mb, a 454 Titanium standard library which generated 539,626 reads, and three paired-end 454 libraries with an average insert size of 2 to 3 kb, which generated 341,814 reads, totaling 269.3 Mb of 454 data. Processes and protocols of library construction and sequencing can be found at http://www.jgi.doe.gov/. The 454 Titanium single-end data and paired-end data were assembled together with Newbler, version 2.6 (20110517_1502). Newbler consensus sequences were computationally shredded into 2-kb overlapping fake reads (shreds). Illumina sequencing data were assembled with VELVET, version 1.0.13 (3), and the consensus sequences were computationally shredded into 1.5-kb shreds. We integrated the shreds and the read pairs in the 454 paired-end library using parallel Phrap, version 1.080812 (High Performance Software, LLC). Gaps between contigs were closed by editing in Consed (4–6) and by PCR and primer walks. Illumina data were used to correct potential base errors and increase consensus quality using the software Polisher (A. Lapidus, unpublished data). Possible misassemblies were corrected using gapResolution (C. Han, unpublished data) or Dupfinisher (7). The final assembly is based on 142.3 Mb of 454 draft data providing 26.9× coverage of the genome and 5,331.6 Mb of Illumina draft data providing 1,006× coverage of the genome.

Annotation was added by the NCBI Prokaryotic Genomes Automatic Annotation Pipeline Group. The 6.33-Mb genome of HD-789 contains 7 replicons: a circular chromosome (5,495,278 bp), containing 5,697 predicted genes, and six circular plasmids. These plasmids contain a total of 929 predicted genes. The G+C content of the chromosome is 35.26%, and the G+C contents of the plasmids range from 33.05% to 39.74% (Table 1). The HD-789 genome contains 121 tRNA and 42 rRNA operons, identified using tRNAscan-SE and RNAmmer, respectively (8, 9). *Bacillus thuringiensis* toxin genes were identified using the Bt_toxin_scanner tool, which integrates BLAST, HMM, and SVM prediction modules (http://bcam.hzaubmb.org/BtToxin_scanner/index.php) (10). Parasporal crystal genes are confined to a single plasmid. Plasmid pBTHD789-3 was found to harbor 7 insecticidal crystal genes, a Cry4Aa3 gene (BTF1_32366), two Cry4Ba5 genes (BTF1_32046 and BTF1_32391), and genes for Cry10Aa3 (BTF1_32386), Cry11Aa3 (BTF1_32101), Cry60Ba3 (BTF1_31831), and Cry60Aa3 (BTF1_31826), plus 3 Cyt toxin genes (BTF1_32411, BTF1_32051, and BTF1_32111) and 1 hemagglutinin gene (BTF1_32201).

**TABLE 1:** Sequence features and accession numbers of replicons from *Bacillus thuringiensis* strain HD-789

Replicon	Accession no.	Length (bp)	% G+C content	No. of:				Total no. of genes	% coding
				Coding sequences	rRNA genes	tRNA genes	Pseudogenes		
Chromosome	CP003763	5,495,278	35.26	5,551	42	104	0	5,697	84.00
pBTHD789-1	CP003764	349,599	33.35	434	0	0	0	434	86.54
pBTHD789-2	CP003765	235,425	36.59	240	0	17	0	257	87.92
pBTHD789-3	CP003766	224,872	33.05	203	0	0	1	204	70.15
pBTHD789-4	CP003767	14,935	39.74	22	0	0	0	22	86.15
pBTHD789-5	CP003768	7,697	35.26	8	0	0	0	8	65.29
pBTHD789-6	CP003769	6,824	35.99	4	0	0	0	4	50.50
Total		6,334,630		6,462	42	121	1	6,626	83.74

*B. thuringiensis* HD-789 is among 53 *B. thuringiensis* isolates representing six different serotypes from branch A of the phylogenetic tree of *Bacillus cereus* group strains based on amplified fragment length polymorphism (AFLP) analysis (11). The most closely related isolates based on AFLP analysis include *B. thuringiensis* HD-795 and *B. thuringiensis* HD-658. Among strains with sequenced genomes, the most closely related strains based on genomic BLAST are *Bacillus thuringiensis* serovar israelensis ATCC 35646 and *Bacillus thuringiensis* IBL 4222.

### Nucleotide sequence accession numbers.

The sequence of the *Bacillus thuringiensis* strain HD-789 has been deposited in GenBank. The accession numbers are listed in Table 1.
